# Editorial: Recent advances and product opportunities in the technology of proteins, probiotics, and prebiotics

**DOI:** 10.3389/fnut.2023.1126929

**Published:** 2023-01-26

**Authors:** Caili Fu, Xi Yu, Hongwei Guo, Chunping You, Jun Du

**Affiliations:** ^1^Food Science and Technology, National University of Singapore (Suzhou) Research Institute, Suzhou, Jiangsu, China; ^2^Faculty of Medicine, Macau University of Science and Technology, Macau SAR, China; ^3^School of Public Health, Fudan University, Shanghai, China; ^4^Key Laboratory of Dairy Biotechnology, Shanghai, China; ^5^Nutrilite Health Institute, Shanghai, China

**Keywords:** protein, peptide, probiotic, prebiotic, personalized nutrition

This special issue, titled “*Recent Advances and Product Opportunities in the Technology of Proteins, Probiotics, and Prebiotics*,” draws together a collection of papers that address current advances in product changes and in the technologies of proteins, prebiotics, and probiotics. Many publications focused on this topic have been reviewed and published that relate to the development of personalized nutrition, which differs from the population-based nutrition intervention that provides one-size-fits-all treatments. Based on the individual's unique characteristics, including anthropometric characteristics, biomarkers, genotypes, gut microbial composition, pre-interventional dietary patterns, and physical activity status, personalized nutrition enables tailored healthy lifestyle choices and thus, improves health outcomes.

Kan et al. explored the potential variables that might alter an individual's response to a specific diet, and the results were transposed into three review articles (Wan et al.; Wang et al.). The gut microbiome, genotype, and phenotypic related biomarkers were specifically highlighted as the most important dimensions for personalized nutrition to achieve its desired goal (Wang et al.). Variations in these dimensions were associated with distinctive nutrition-related traits, including the bioaccessibility, bioavailability, and utilization of nutrients, which subsequently, affected the efficacy of nutritional intervention (Kan, Wu, et al.; Wan et al.). Building upon these endeavors, this group of researchers performed the first personalized nutrition study in China to test the hypothesis that the deployment of the above-mentioned dimensions in a nutritional intervention study could result in a greater lifestyle change among obese adults (Kan, Yi, et al.; Zheng et al.). Their theory was supported by the subjects' much greater decreases in body mass index, waist measurement, and percentage of body fat in the personalized nutrition-treated team compared to the standard study participants (Kan, Yi, et al.). In a subsequent investigation, Zheng et al. identified the gut microbiome's role in modifying lipid metabolism outcomes connected to fat distribution and obesity-related gene polymorphisms, while Zhang et al. discovered that through altering the gut microbiota, *Lactiplantibacillus plantarum* ST-III-fermented milk can ameliorate autistic-like symptoms of mice with autism spectrum conditions brought upon by the use of valproic acid. Zheng et al. summarized the systematic assessment of the prebiotics' contribution to promoting probiotics (You et al.). By identifying novel ligand fishing models using nanotechnologies for obesity treatment, Tian et al. showcased another method to promote further advancements in personalized nutritional interventions. Noticeably, machine learning was utilized to capture complex relationships between the phenotypic, genomic, and metagenomic features and nutritional needs of an individual to develop tailored dietary and nutritional advice. The fact that computational algorithms need to be trained by big datasets will add tremendous value to multi-omics approaches (Wang et al.). Some interesting works that involved the novel utilization of natural products and microorganisms are also included. Wang et al. successfully used Lactobacillus for the improvement of meat quality in Sunit sheep. And they further revealed that the underlying mechanism of such improvement is related to the altered mitochondrial biosynthesis via the AMPK pathway. Wu et al. investigated and found that by targeting ferroptosis, traditional Chinese medicine has a preventive impact on non-alcoholic fatty liver disease and liver cancer.

## Author contributions

CF and XY researched the relevant literature and created the initial version of the draft. HG, CY, and JD amended the manuscript. All of the contributors have reviewed and consented to the published version of the manuscript.

